# Social Interactions in Myxobacterial Swarming

**DOI:** 10.1371/journal.pcbi.0030253

**Published:** 2007-12-28

**Authors:** Yilin Wu, Yi Jiang, Dale Kaiser, Mark Alber

**Affiliations:** 1 Department of Physics, University of Notre Dame, Notre Dame, Indiana, United States of America; 2 Center for the Study of Biocomplexity, University of Notre Dame, Notre Dame, Indiana, United States of America; 3 Theoretical Division, Los Alamos National Laboratory, Los Alamos, New Mexico, United States of America; 4 Department of Biochemistry, Stanford University, Stanford, California, United States of America; 5 Department of Mathematics, University of Notre Dame, Notre Dame, Indiana, United States of America; University of Texas at Austin, United States of America

## Abstract

Swarming, a collective motion of many thousands of cells, produces colonies that rapidly spread over surfaces. In this paper, we introduce a cell-based model to study how interactions between neighboring cells facilitate swarming. We chose to study *Myxococcus xanthus,* a species of myxobacteria, because it swarms rapidly and has well-defined cell–cell interactions mediated by type IV pili and by slime trails. The aim of this paper is to test whether the cell contact interactions, which are inherent in pili-based S motility and slime-based A motility, are sufficient to explain the observed expansion of wild-type swarms. The simulations yield a constant rate of swarm expansion, which has been observed experimentally. Also, the model is able to quantify the contributions of S motility and A motility to swarming. Some pathogenic bacteria spread over infected tissue by swarming. The model described here may shed some light on their colonization process.

## Author Summary

Many bacteria are able to spread rapidly over the surface using a strategy called swarming. When the cells cover a surface at high density and compete with each other for nutrients, swarming permits them to maintain rapid growth at the swarm edge. Swarming with flagella has been investigated for many years, and much has been learned about its regulation. Nevertheless, its choreography, which is somewhat related to the counterflow of pedestrians on a city sidewalk, has remained elusive. It is the bacterial equivalent of dancing toward the exit in a crowd of moving bodies that usually are in close contact. Myxococcus xanthus expands its swarms at 1.6 μm/min, about a third the speed of individual cells gliding over the same surface. Each cell has pilus engines at its front end and slime secretion engines at its rear. Using the known mechanics of these engines and the ways they are coordinated, we have developed a cell-based model to study the role of social interactions in bacterial swarming. The model is able to quantify the contributions of individual motility engines to swarming. It also shows that microscopic social interactions help to form the ordered collective motion observed in swarms.

## Introduction

Bacterial swarming, a coordinated motion of many bacterial cells, facilitates their spread on the surface of a solid medium, like agar [1]. Swarming may have evolved to permit the bacteria in a colony to expand their access to nutrients from the subsurface and to oxygen from above. When the surface is a tissue in a live host, pathogenic bacteria swarm to create a biofilm and to spread the infection. Swarming is observed in cells that are propelled by rotating flagella [2], by the secretion of slime [3], and by retracting type IV pili [4,5]. Bacterial swarming has been studied quantitatively in the modeling context of self-propelled particle systems [6–8]. Most models, such as those for Bacillus subtilis and Escherichia coli (see [8] for a review), are based on long-range cellular interactions facilitated by chemical gradient or nutrient level (chemotaxis). However, myxobacteria show no evidence of long-range communicating systems to guide their collective motion; they have only local contact signaling and use social interactions between neighboring cells for swarming [9]. How interactions between cells facilitate swarming is still an open question. Understanding this question might shed light on the self-organizing process in bacteria, such as the spreading of a biofilm in an infected tissue and the development of multicellular fruiting bodies [4,5]. In this paper, we describe a new cell-based model and study the effects of social interactions between cells, including the interaction mediated by slime trails and by type IV pili, on swarming. Type IV pili are found at one pole of a wide range of bacteria, including many pathogens that cause plant and animal disease.

We chose to examine Myxococcus xanthus because it swarms rapidly, has typical type IV pilus engines at the front end of cells, has slime secretion engines at the rear, and coordinates the two engines with each other. M. xanthus has been studied for more than a century; numerous swarming mutants have been identified and characterized. Myxobacteria are commonly found in cultivated soils, where they feed on other bacteria. On the surface of nutrient agar, they swarm away from a point inoculum, spreading outward at a constant rate for 2 wk. Although the bacteria are growing (and in fact they must grow to swarm), 90% of the swarm expansion rate is due to motility and to interactions associated with motility, as shown by the low spreading rate of nonmotile mutants [10,11].

Individual M. xanthus cells are rod shaped, roughly 5 μm in length and 0.5 μm in width. They have two types of molecular motors that provide the thrust necessary for their gliding movement over a surface [9]. At the leading end of the cell are retractile type IV pili, long and thin hairs responsible for S motility. When a cell is close to a group of other cells, the cell's type IV pili can attach to the fibrils, which cover the surface of the neighboring group of cells like a fisherman's net. After attachment, the pilus retracts, and the retraction force pulls the piliated cell forward, while the group hardly moves. This pilus-mediated interaction produces many asymmetric cell clusters that often have tips that are pointed at one end (arrowhead-shaped) and is characteristic of S motility. Arrowheads can be seen in the young swarms of A^−^S^+^ mutants [11]. S motility is found among pathogenic *Neisseria* and *Pseudomonas,* where it is called twitching motility [4,5].

At the trailing end of myxobacterial cells are several hundred pores, from which slime is secreted. There are roughly 150 pores scattered over the sides of the cell, also secreting slime that becomes a thin layer, protecting the cell from lysis by cell-wall digestive enzymes being secreted by all the cells [3]. Both the lateral and the polar slimes are thought to be the same polysaccharide that is part of A motility (hereafter simply referred to as slime). Importantly, slime is completely distinct from the fibril polysaccharide that serves S motility [11]. Slime secretion from the rear pushes the cell forward, leaving a trail of slime behind the cell [3,12] and generating movements called A motility. When a moving cell encounters a slime trail, it tends to turn through the acute angle to follow the slime trail. When an A motile cell collides with the side of another cell, the pushing of the slime engines at the rear causes the cell, which is flexible, to bend. The colliding cell thus reorients parallel to the other cell, producing a side-by-side cluster of cells. Such clusters are transient because the two cells do not adhere and often slide past one another.

The A and S motility engines, which are located at opposite poles of the rod-shaped cells, have engine-specific social interactions. During movement, a cell's polarity reverses regularly every 10 min or so [13,14], and reversal is required for swarming [15,16].

**Figure 1:**
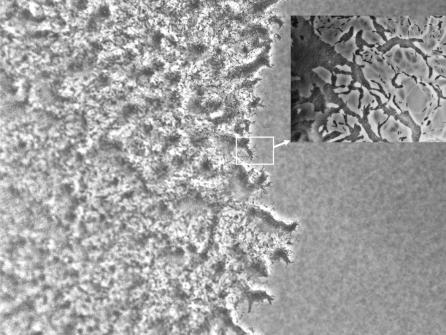
Picture of a Rectangular Section of a Typical Swarm Edge of Wild-Type M. xanthus Strain DK 1622 (A^+^S^+^) Several small peninsulas project outward from the edge of the swarm. The inset is a higher-magnification view of a segment of a typical swarming edge in which single cells and clusters of cells are evident. The inset was taken from a different but similar experiment. Dense clusters of cells (darker shades of gray) are evident in both pictures.

**Figure 2:**
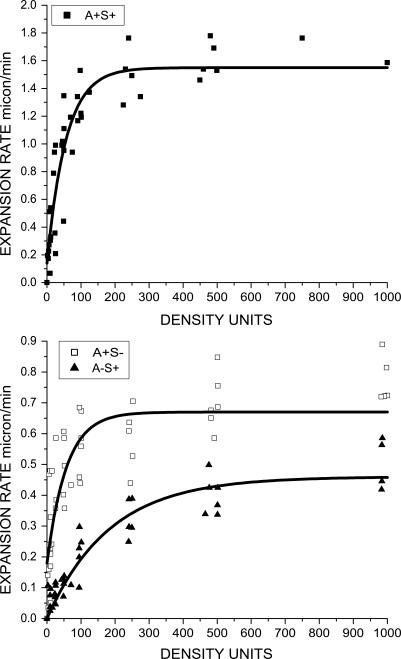
Fitting Curves of Spreading Rates of Wild-Type (A^+^S^+^) Myxobacteria and Motility Mutants (Reproduced by Using Data from [10]) The dots are experimental data points. The fitting functions are as follows: wild-type (A^+^S^+^), *f*(*x*) = *a* − *b* × exp(−*x* / *c*), with *a* = 1.55 ± 0.06, *b* = 1.41 ± 0.10, and *c* = 56 ± 10; A^+^S^−^ mutant, *g*(*x*) = *a* − *b* × exp(−*x* / *c*), with *a* = 0.67 ± 0.03, *b* = 0.49 ± 0.05, and *c* = 57 ± 16; and A^−^S^+^ mutant, *h*(*x*) = *b* × (1 − exp(−*x* / *c*)), with *b* = 0.46 ± 0.02 and c = 184 ± 27. The density is in K-S units, and the expansion rate is in microns per minute.

A wild-type cell (A^+^S^+^) expresses both A and S motilities. A^+^S^−^ mutants express only A motility, while those with S motility but no A motility are called A^−^S^+^ mutants [9]. Because wild-type and A^+^S^−^ mutants are self-propelled by A motility engines, a comparison can expose the social interactions specific to the type IV pili. In both cases, individual cells are observed to move, stop, and move again, sometimes slightly changing direction and regularly reversing [3]. To investigate the coordinated motion within M. xanthus swarms, culture droplets of each mutant were placed on agar plates, and the swarm expansion rates were measured [10]. Figure 1 shows the edge of a typical swarm of wild-type (A^+^S^+^) cells. It is observed that swarm expansion rates remain constant until the swarm covers the entire surface available [10]. The expansion rates for various initial cell densities in K-S units were measured and plotted against the cell densities. (K-S is Klett-Summerson unit; a measurement of cell density in suspensions [10]. A sample of cell suspension with 100 K-S units has approximately 4 ×10^8^ cells/ml. Using the experimental data in [10], we find that 100 K-S units correspond to a close-packing arrangement of cells in a 2-D area.) The fitted functions of expansion rate data for the three cell types are shown as solid lines in Figure 2. To a first approximation, the velocity of individual cells, when they are moving, is the same for S^−^ mutants (A^+^S^−^) and wild-type (A^+^S^+^) cells, about 4 μm/min, but their swarm expansion rates are different [10]. The A^+^S^−^ and A^−^S^+^ mutants swarm with a maximum rate of 0.67 μm/min and 0.46 μm/min, respectively. Surprisingly, when S motility cooperates with A motility in wild-type M. xanthus (A^+^S^+^), the maximum swarming rate is 1.55 μm/min, about 2.3-fold larger than that of A^+^S^−^ ([10], as shown in Figure 2).

Previously, we used a lattice-based model to study myxobacterial fruiting body development after starvation [17,18]. Swarming with sufficient nutrient supply has been studied using a continuous model in the form of partial differential equations (PDEs) [19]. The effects of engine mechanics and cell shape have yet to be taken into account. Recently, we introduced a simplified off-lattice stochastic description of swarming [20], and herein add our current understanding of engine mechanics to investigate swarming and the role of social interactions.

This paper is organized as follows. We start by describing the model of cell behavior and social interactions. Then, we present the simulation results and compare them with the experimental observations. We demonstrate a constant rate of swarm expansion and show that the model accounts for the significant difference in swarming rates between wild-type and A^+^S^−^ myxobacteria arising from the loss of S motility. We also study in detail the order of collective motion in myxobacterial swarms. A detailed description of the computational model is given in the Methods section.

## Results

### Model of Cell Behavior and Social Interactions

**Figure 3:**
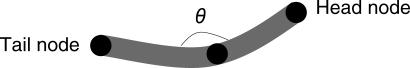
The Cell Body of a Bent Cell Is Represented by Three Nodes (Solid Black Dots) The cell has a length-to-width ratio of 10:1.

In this paper, we focus on the collective motion of a large number of cells in a swarm of high cell density, taking only the local, contact-mediated interactions between cells into account. We represent each cell as a string of *N* nodes in a 2-D space, following our earlier work [20] (Figure 3). The vector pointing in the direction from the tail node to the head node represents the orientation of a cell. We define an energy function (Hamiltonian) for the node configuration of a cell body and use it to constrain the cell length and the cell shape to a certain range. The active motion of an individual cell is then modeled as follows. After the head moves in a particular direction, a Monte Carlo approach [21] is used to reconfigure positions of other nodes in an attempt to minimize the Hamiltonian (see Methods). This allows the cell body to bend and to change its length by random fluctuations, which reflects the experimental observations [22].

As mentioned in the introduction, the measured velocities of individual cells vary over a wide range, but the average velocities of A^+^S^−^ and A^+^S^+^ cell types are similar. To a first approximation, we take the cell velocity to be constant and the same for wild-type A^+^S^+^ and A^+^S^−^ cells, with a magnitude of 4 μm/min [10]. The direction of cell movement is determined dynamically by the model, which takes the interactions between neighboring cells into account.

Frequently cells reverse their motion by 180°. Reversals are regulated by an internal biochemical clock that is not affected by collisions or other interactions between cells [15,16]. We model regular reversals of cell motility engines by switching roles of head and tail nodes in accordance with an internal clock (see Methods). The swarming efficiency (the ratio of the swarm expansion rate to the speed of individual cells) of myxobacteria primarily depends on social interactions between neighboring cells. The expansion rate of a swarm without social interactions would be zero, since the cells would move back and forth equally without any net displacement in the long run. Social interactions help a swarm of reversing cells to spread.

### Social Interactions between Neighboring Myxobacterial Cells

Ideally, interactions between all the more than 10^7^ cells in a swarm would be considered, but that is not possible in practise. Instead, we try to identify for each cell a neighborhood within which a majority of its interactions are expected to be found. Social interactions arise in S motility when the type IV pili of one cell attach to the fibrils that surround other cells. Social interactions arise in A motility from the tendency of a cell to follow the trail of slime left by another cell, and from collisions between cells that cause a moving cell to stop and its engines to stall, or those cause a cell to change its direction. Using the experimental data of Figure 2, an area of interaction for each cell type, A^+^S^−^, A^−^S^+^, or A^+^S^+^, was defined as the statistically averaged area around a cell within which most of its social interactions occur. The interaction areas were taken to be proportional to the inverse of parameters in the exponential term of the formula obtained when an exponential curve was fitted to the experimental data in Figure 2. Fitting functions are specified in the legend to Figure 2. Each curve represents the observed swarm expansion rate as a function of the initial cell density of the culture. The interaction area for wild-type cells was found to be smaller than the sum of the interaction areas of the A^+^S^−^ and A^−^S^+^ mutants. We suggest that this unanticipated finding results when both engines are working because the two engines on a wild-type cell are not statistically independent but are constrained by the structure of a cell to propel it in the same direction.

**Figure 4:**
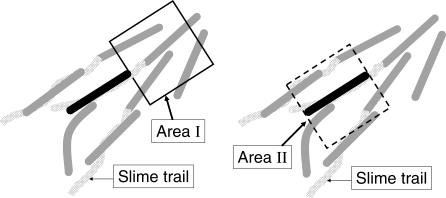
Diagram Showing the Two Types of Social Interactions for a Cell (Black) Although the pilus length varies with extension, retraction, and breakage, most pili are on the order of one cell length [39]. Area I represents the pilus–cell interaction area. Its sides are taken as the average pilus length. If either the head or the tail of another cell falls within this area, it can be contacted by pili from the black cell*.* Area II is the corresponding interaction area for A motility. A bent gray cell in direct contact with a dark cell illustrates the bending and alignment due to collisions between cells. Slime trail following is illustrated by trails (light gray shaded area) inside of area II. An artificially low cell density has been used in this figure to clarify the several interactions. In reality, many cells are adjacent to each other within the interaction area.

Pilus-mediated interactions depend on the dynamics of pilus retraction [23] and on the spatial distribution of the fibrils to which the pilus tips have attached [24,25]. Although these factors are mechanically complex and not yet understood in detail, the interaction has straightforward effects. Pilus retraction provides a driving force for cell movement that happens to be large, several times larger than the force developed by muscle acto-myosin. And, because the force is almost never directed along the cell's long axis, the force tends to reorient the direction of gliding. Because we are confined by the approximation that isolated cells move with constant speed, we need only consider the reorienting effect of pilus retraction. No effect on cell speed is considered, except that it drops to zero when one cell collides with another. Inasmuch as the fibrils tend to bundle groups of cells, as will be described below, the large size of the cell cluster prevents a significant reorientation of the bundle; only the cell whose pili have attached is reoriented. We model the reorientation effect of pilus-mediated interactions as driving the local alignment of cells (see area I of Figure 4 and Methods). Although we represent the interaction area by a rectangle, a circle or some irregular domain could have been used. The important quality of an interaction domain is its area. That area is proportional to the probability that a cell has an interaction. Swarms of wild-type cells cover a larger area than those of A^+^S^−^ or A^−^S^+^ mutants [11]. Moreover, the peninsulas are denser with cells that are well-aligned side by side [10]. Both effects illustrate reorientation due to pilus retraction. Cell clusters tend to be narrow in the case of an A^+^S^−^ mutant and wide in the case of wild-type bacteria.

A motility engines at the rear of the cell push it forward in the direction of their long axis. A motility also produces slime trails, and cells tend to follow them due to the adhesion of newly secreted slime to the older slime in the trail. The resulting alignment of the slime polysaccharide chains also reorients the direction of gliding. Slime trails are represented in the model by the paths that were taken by the last cells to have passed through area II (Figure 4). Further details are given in the slime orientation field described in Methods. Rod-shaped A motile cells, which are pushing at their tail ends, tend to form parallel arrays if they collide or come into close contact with each other. These effects are illustrated in area II of Figure 4 and are elaborated in Methods. Alignment results from inelastic collisions between cells that change their orientations. More generally, alignment in regions of high cell density arises spontaneously from the physical clustering of self-propelled rods [26].

For wild-type cells, we first model A and S motilities, individually. Then, we combine them under the approximation that isolated cells move at a constant rate, as described above. The persistent active motion is taken to be led by the head of the cell, no matter which engines are functioning. Finally, we model the reorientation due to pilus retraction and to the alignment of A motile cells with their neighbors, or with the slime field.

### Swarm Expansion

**Figure 5:**
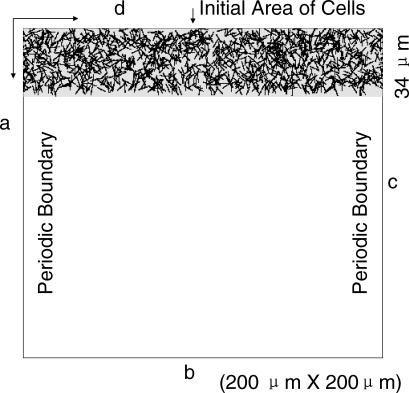
The Simulation Domain Periodic boundary conditions are used at sides a and c, while reflection boundary condition is imposed at side d (see Methods). Cells at a density of 50 K-S units are shown randomly distributed in the initial area. Their initial orientations are chosen in accordance with the distribution function described in Methods, Equation 12.

To test the consistency of the model, we simulated the motion of cells near the edge of the swarm, and studied the expansion of the swarm. Although M. xanthus swarms consist of many millions of cells, the radial symmetry of a swarm makes it possible to consider a small rectangular sector of the swarm (Figure 1). A rectangular area of 200 μm by 200 μm (Figure 5) was convenient.

To compare the simulation with experimental measurements (shown in Figure 2), we considered that growth in the center of the swarm was driving a net radial outflow of cells from their center [15], and that the swarm was expanding at a constant rate. A constant cell density near the swarm edge was observed experimentally as the edge moved out [10]. Skipping the early transient phases, we start the simulation after the steady state has been reached. Although cells in the initial area are oriented in all directions, the orientations are radially symmetric. Both conditions apply to the “Initial Area of Cells” in Figure 5.

Denoting cell density as *p*(*r*, *θ*) and the radial density as *P*(*r*), due to the symmetry and the steady state, we have:

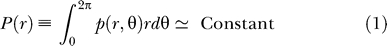



**Figure 6:**
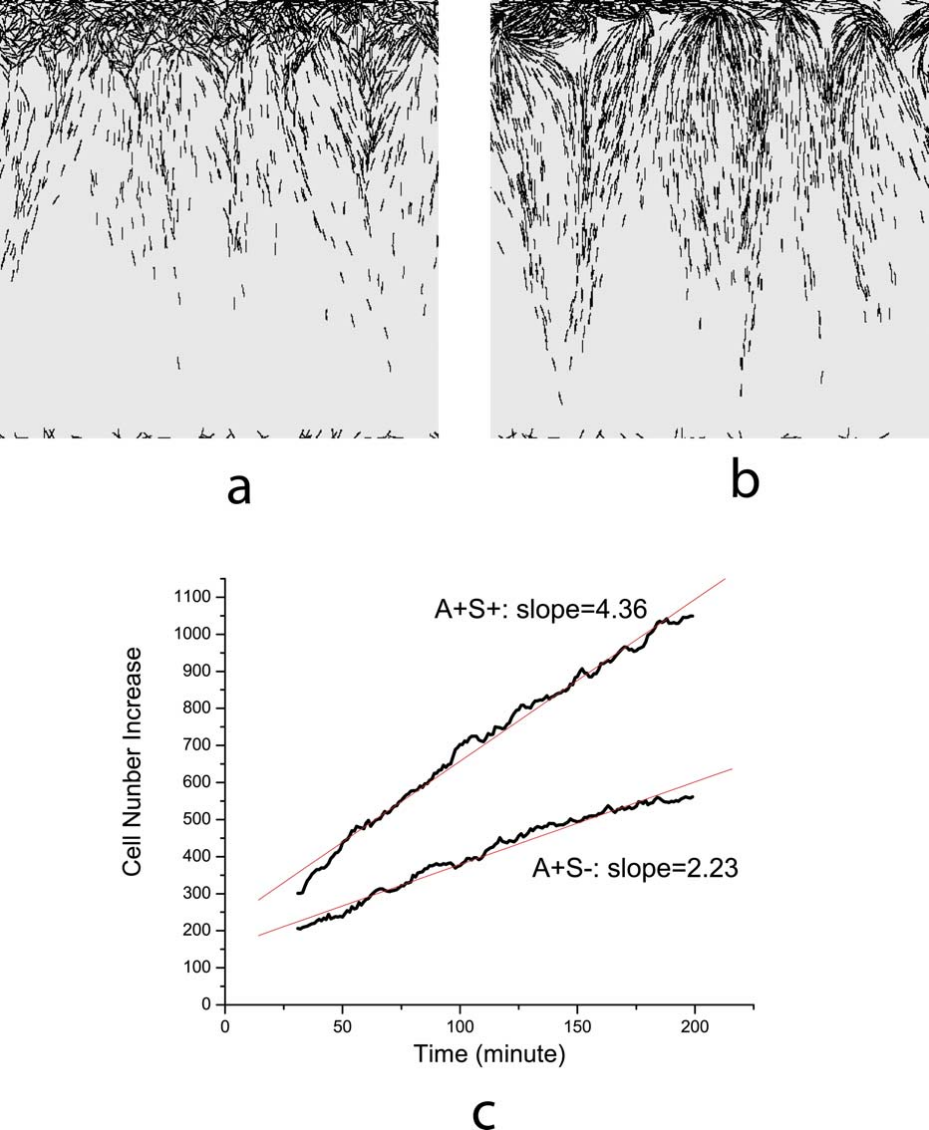
Simulation Images and Linear Growth of Colony (A) and (B) are pictures of the edges of the A^+^S^−^ and wild-type (A^+^S^+^) swarms, respectively, after 200 min of simulation. (C) Linear increase in the number of cells in the simulation domain with time. The red lines are best fits of simulation data with slopes indicated in the plot.

This relation shows that the cell number flux across the lower boundary of the initial area (or the increase rate of total cell number in the whole simulation domain) is linearly correlated with the colony expansion rate in Figure 2. We calculate the cell number flux rather than expansion rate directly. Therefore, we do not have to increase the simulation domain or the total number of simulated cells. Further details of the simulation setup, implementation of the algorithm, and the choices of parameters are described in Methods and Table 1. Simulations show formation of long clusters (peninsulas) in both A^+^S^+^ and A^+^S^−^ cases (see Figure 6A and 6B), which was observed experimentally [10].

**Table 1:**
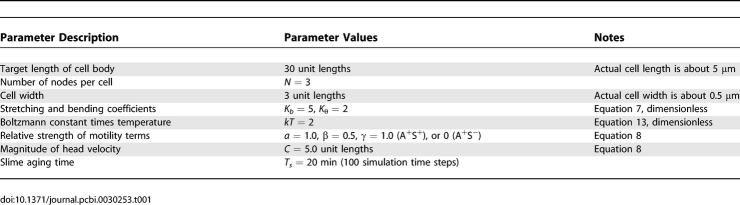
Parameter Values Used in the Simulations

Simulations were performed for cell densities ranging from 2 to 200 K-S units, and linear increase of cell number was observed in all cases (for example, see Figure 6C). This implies that the cell number flux is almost constant during the whole swarming process for a given initial cell density, in full agreement with experiments [10]. Figure 6 corresponds to an initial density of 50 K-S units, which is a near saturation density for the rate curves of Figure 2.

### Comparison of A^+^S^+^ and A^+^S^−^ Swarm Rates

**Figure 7:**
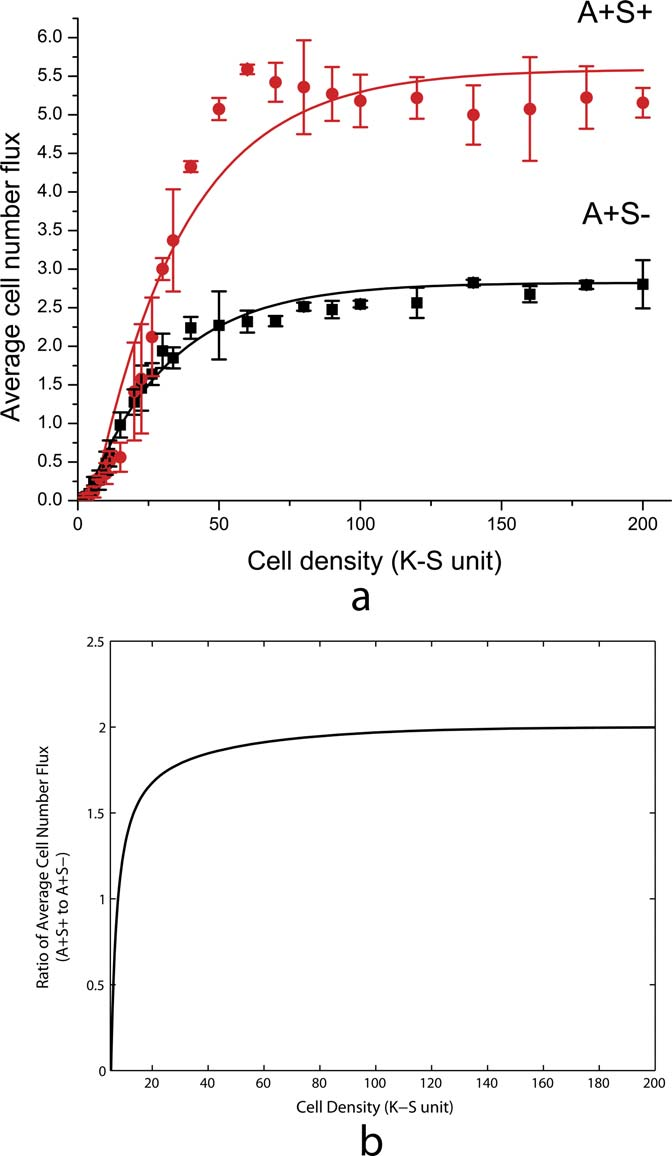
Average Cell Number Flux of Wild-Type and A^+^S^−^ Mutants (A) The average cell number flux data from three simulation runs, with error bars indicating standard deviation. The red dots and black squares indicate results for wild-type cells and A^+^S^−^ mutants, respectively. (B) Plot of the ratio of fitting functions *f*(*x*) / *g*(*x*) against cell density.

We have calculated linear fits for the cell number increase data at various cell densities, and taken the slopes to be the average cell number fluxes, as shown in Figure 6C. The results for both A^+^S^+^ and A^+^S^−^ cells are plotted against cell density in Figure 7A. We found that the cell number flux of the wild-type cells (A^+^S^+^) is greater than that of the A^+^S^−^ mutant at all cell densities. At densities higher than 50 K-S units, the cell number flux for A^+^S^+^ is 2-fold larger than the A^+^S^−^.

To see this effect more clearly, we fit the average cell number flux data into the first order exponential decay function, which is similar to the function used in Figure 2 from [10]. The fitting functions for wild-type A^+^S^+^ cells and A^+^S^−^ mutants are found to be *f*(*x*) = 5.6 − 6.6 × exp(−*x* / 32.1) and *g*(*x*) = 2.8 − 3.1 × exp(−*x* / 29.4), respectively. The ratio of these two fitting functions is plotted against cell densities in Figure 7B. It is equivalent to the ratio of colony expansion rates since the cell number flux is linearly correlated with the expansion rate. The ratio first increases and then saturates around 2 for cell densities higher than 50 K-S units. Experimental data shows that the A^+^S^+^ rates are 2-fold to 2.5-fold larger at cell densities higher than 50 K-S units (Figure 2). Therefore, our result shows a significant difference in swarming rates between wild-type and A^+^S^−^, arising from the contribution of S motility that agrees with the experiment.

Collision occurs in the model whenever the head of one cell overlaps the area occupied by another cell. At this point, the moving cell stops; it is not permitted to glide on top of the other cell. As a consequence, at high cell densities the movement of individual cells is reduced. In reality, cells do glide over each other. Reduction becomes significant above 100 K-S units, because at 100 K-S units the average area occupied by an individual cell is close to the area of a cell body (i.e., the area is closely packed with cells). In practice, due to the tendency of cells to cluster, cell movement is reduced beginning at concentrations of 60 K-S units. This effect explains the decrease in cell flux observed at higher cell densities (>60 K-S units) for the wild-type cells in Figure 7A. The decrease results in a smaller value of maximum ratio (about 2-fold; see Figure 7B) than experimental data (about 2-fold to 2.5-fold).

### The Swarming of A^−^S^+^ Mutants

Comparing the three curves of Figure 2 shows clearly that S motility contributes to the swarming of wild-type (A^+^S^+^) cells. Figure 2 also shows that A^−^S^+^ swarms expand without help from A motility, although the rate of expansion is less than one-third that of the wild-type cells at every cell density. With these data in mind, a puzzle takes shape: how are pili able to support expansion of an A^−^S^+^ swarm when there should be no fibrils to which the type IV pili might attach beyond the edge of the swarm? The surface ahead of the swarm edge never had cells upon it. Must the belief that pili attach to fibrils before they can retract be abandoned? This section describes an attempt to solve the puzzle by examining the evidence that pili bind fibrils specifically, by offering a mechanism whereby specific binding and retraction can bring about the expansion of an A^−^S^+^ swarm, and by testing the mechanism proposed.

Evidence for specific binding includes the observation that A^−^S^+^ cells move only when they are within a pilus length of another cell [10,27]. Fibrils are present in profusion, and they envelope clusters of adjacent cells (see Figure 2 from [28]). Although only half of the fibril mass is polysaccharide (the other half is protein [24]), several experiments have revealed that removing the protein has no effect on pilus binding [24,25,29–31]. Evidently, M. xanthus pili bind fibril polysaccharide.

**Figure 8:**
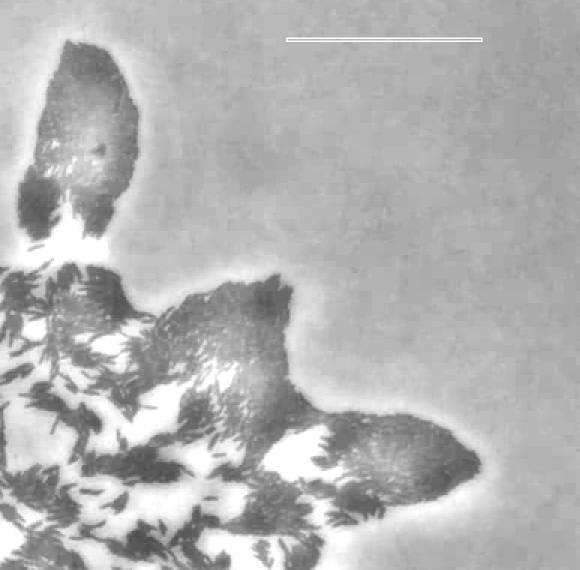
Phase-Contrast Image of the Cell Distribution at the Edge of a Young A^−^S^+^ Swarm That Is Expanding to the Right Older A^−^S^+^ swarms stop expanding when their edge becomes smooth and many layers deep. The white scale bar is 50 μm long.

Therefore, side-by-side clusters of M. xanthus cells, like the peninsulas in Figure 8, are viewed as a bundle that is enveloped by an elastic fisherman's net formed by association of polysaccharide fibrils that the cells have secreted. Bundling of cells by fibrils offers an explanation for the pointed shape, which A^−^S^+^ peninsulas tend to have. The points aim away from the swarm center (Figure 8 and [10,32]) and in the general direction of swarm expansion. The shape and orientation of the peninsula tips suggest that cells at the tip of the peninsula have been pushed into their position at the tip. Consider a cell within the body of the peninsula that happens to be moving toward the tip of the peninsula. This cell will have projected its pilus forward and attached it to the fibril network on cells ahead of it and closer to the tip. Retraction of that pilus could pull the cell forward and upward to add a new layer of cells to the peninsula. Indeed, most peninsulas have a second (or third) layer near their tips, which are evident in Figure 8. On other occasions, retraction would pull the piliated cell right up to the end of a cell in the bottom layer that lies just ahead of our piliated cell. Recalling the description of A motility in the Introduction, each cell is also covered by the slime polysaccharide, which protects them from autolysis. Since the network of fibrils that envelops cells of a peninsula bundles them, both the elasticity of the fibrils and the cohesion between the slime on adjacent cells would tend to prevent their separation, by wedging action of the rounded end of the pushing cell, from cells to their left and right in the tip of the peninsula. Consequently, complete retraction of the pilus would cause the moving cell to push the cell in the peninsula that is immediately ahead of it. The pushed cell might slide forward while adhering through its slime covering to the cells on either side. Localized sliding would be reflected in a sharpening of the tip contour to a point, as observed (Figure 8).

The hypothesis of pushing by S motile cells was tested by analysis of seven time-lapse movies of the advancing edges of A^−^S^+^ swarms, each movie of 1 h to 3 h in duration. Figure 8 is a single frame from one of the movies. In that frame, numerous single cells and many peninsulas of various sizes are evident. Several observations relevant to A^−^S^+^ swarming could be made from the movies (Key and Kaiser, unpublished data). First, almost all of the many thousands of cell movements were found within clusters of ten or more cells. No isolated cell moved significantly, unless the cell was within pilus-striking distance of another cell. This shows that the cells are moving with S motility alone. Second, although the peninsulas either elongated or moved forward, the translocation rate was much less than the rate of individual cell movement in the same field. A lower rate correlates the peninsula's advance to its being pushed from behind, because the hypothesis has the pushed cell sliding past its neighbors in the peninsula. The sliding friction would decrease the rate of advance. Finally, the movies show many examples of individual cells, which appear to be moving more or less randomly, behind an arrowhead or a peninsula. Individual cells advancing toward the rear edge of the peninsula could have pushed it. A quantitative analysis of cell movement in the movies will be published separately, but this qualitative analysis supports pushing.

### Social Interactions Result in Higher Order of Collective Motion

In previous sections, we have shown that our model for social interactions is consistent with experimental results at the level of individual cells. In this section, we investigate how microscopic social interactions facilitate swarming at the population level. We demonstrate that social interactions lead to an increase in the order of collective motion, which is strongly correlated with swarming efficiency. We start by introducing an order parameter to characterize collective motion of bacteria in swarms with complex clustering patterns.

After analyzing experimental data and taking into account regular reversals of myxobacteria cells, we define the most ordered state as follows: all cells move side by side in close contact with each other in the same or opposite direction. The collective motion is considered purely nonordered when either one of the following criteria is satisfied: (1) the orientations of neighboring cells of any given cell are random (or uniformly distributed); and (2) any pair of cells is well-separated so that cells are not in direct contact.

Vicsek et al. [33] used the average velocity as a global order parameter for analyzing the motion of self-propelled particles. However, myxobacteria cells reverse regularly, and two opposite directions should be considered as being equivalent to each other. There are always cells moving in the direction opposite to the net motion of the whole cluster in most cell clusters in experimental movies. Also, as shown in the inset of Figure 1, the swarming pattern often exhibits localized clusters of aligned cells with different orientations of motion, and one would need to take local order into account when measuring global order of motion. Therefore, the average velocity is not the best way of measuring the nematic order in myxobacteria swarms.

**Figure 9:**
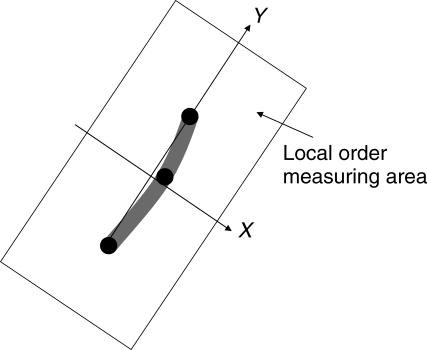
Local Order Measuring Domain

We first define two local measuring components to describe the local orientational order and positional order of a given cell, denoted as Ψ and P, respectively. For a given cell *k* (*k* = 1,2…M, M is the total cell number), we choose the rectangular domain (of area *s*_0_) illustrated in Figure 9 as the local measuring domain (one cell length by two cell lengths), centered at the center of mass of a cell. We then measure the total area *S* occupied by neighboring cells within the local measuring domain and define the local positional order as the following:

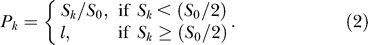



We record the orientations *θ_j_*, with *j* = 1,2,…*n* of the neighboring cells with either head node or tail node inside the local measuring domain, in a way used in Equation 11 in Methods. Then the angles between these orientations and the *x*-axis in Figure 9, 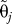 , with *j* = 1,2,…*n*, and 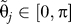 , are calculated. If cell *k* has no neighbors (*j* = 0), we define Ψ as 0. Otherwise, the local orientation order function is defined as follows:

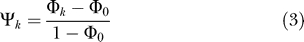
with

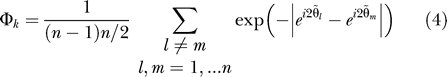
and

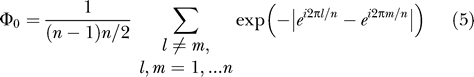



Φ_k_ determines how ordered the distribution of 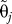 is. The most ordered state corresponds to the case when all 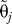 are equal and Φ_k_ has a maximum value of 1. Φ_k_ is rescaled to the range between 0 and 1. Therefore, in the case of uniform (random) distribution of 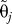 , the local orientational order function Ψ*_k_* is equal to 0. In the most ordered state, all 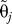 are equal and Ψ*_k_* is equal to 1.

Finally, we combine both the local orientational order and positional order components from Equations 3–5 to define the global order parameter for the collective motion of myxobacteria:

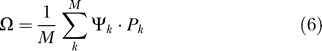
where *M* is the total number of cells.

**Figure 10:**
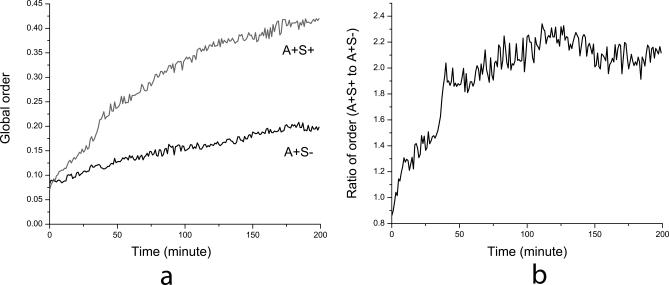
Time Evolution of the Global Order Parameter (A) Plots of the order parameter Ω for the simulation of swarming near colony edge with initial cell density of 50 K-S units. (B) Plot of the ratio of global order parameters for A^+^S^+^ and A^+^S^−^ swarms.

The order parameter Ω has been specifically designed for myxobacterial swarming. Figure 10 shows values of Ω for the simulation of swarming near colony edge with initial cell density of 50 K-S units. We find that the order of collective motion in both A^+^S^+^ and A^+^S^−^ swarms steadily increase, and that A^+^S^+^ cells achieve a much higher (about 2-fold) order than A^+^S^−^ cells.

**Figure 11:**
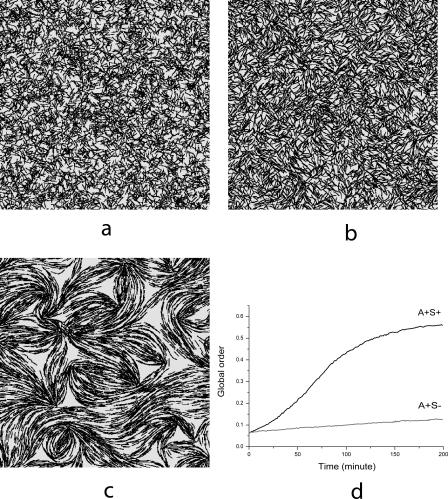
Simulations of Cell Motion Deep Inside the Swarming Colony (A) Initial random distribution of cells in a square area of size 167 μm × 167 μm at the density of 50 K-S units with periodic boundary conditions. (B) A^+^S^−^ mutant swarm after 3 h of evolution. (C) Wild-type (A^+^S^+^) swarm after 3 h of evolution. (D) Plot of the global order parameter Ω for the simulations of wild-type (A^+^S^+^) and A^+^S^−^ mutant swarms.

Further, we look at the order of cellular motion in the inner area of myxobacteria colony. In Figure 11A, cells are randomly distributed in a square area of size 167 μm × 167 μm with a density of 50 K-S units. All boundary conditions are periodic. This is different from the previous simulations for cells near the colony edge, because we do not assume a preorganized orientation distribution of cells.

Figure 11B and 11C are the simulation pictures after 3 h for A^+^S^−^ mutant and wild-type cell (A^+^S^+^) swarms, respectively. We see that the pattern of A^+^S^−^ mutant exhibits lower order, while wild-type (A^+^S^+^) cells form large clusters oriented in various directions. Plots of the parameter Ω are presented in Figure 11D. Again, we see that the order of motion in both A^+^S^+^ and A^+^S^−^ cases increase with time, while A^+^S^+^ cells achieve a much higher order than A^+^S^−^ cells.

Therefore, we demonstrate that social interactions lead to an increase in the order of collective motion. Type IV pilus-mediated interactions increase the order much greater than social interactions associated with A motility. This is consistent with the experimental findings by Pelling et al. [32], who observed higher-order patterns within wild-type (A^+^S^+^) swarms in comparison with motility mutant swarms.

Comparison of Figure 10B with the ratio of cell number fluxes (Figure 7B) indicates that the order of collective motion strongly correlates with the swarming efficiency. We suggest that higher order of motion results in greater swarming rates as observed in wild-type myxobacteria experiments. It explains the origin of the significant difference in swarming rates between wild-type and A^+^S^−^ myxobacteria arising from the coupling of S and A motilities.

## Discussion

We have developed an off-lattice cell-based computational model to study the role of social interactions in bacterial swarming. The model is stochastic and is based on detailed description of the bacterial motility engines and their regulation. The model demonstrates how social interactions facilitate bacterial swarming, and provides an explanation to the significant difference in swarming rates between wild-type and A^+^S^−^ mutants arising from the effects of S motility. Our simulations indicate that the order of collective motion strongly correlates with the swarming efficiency, which provides a connection between microscopic social interactions and population-level swarming behavior.

The model is two-dimensional and provides a very good approximation for the bacterial behavior near the edge of the swarming population. However, in experiments at higher densities, cells were observed to glide on top of each other, resulting in multiple cell layers just behind the edge of the swarm. As discussed in Results, the 2-D nature of our model causes a slight decrease in cell number flux at higher cell densities (>60 K-S units) for wild-type myxobacteria (Figure 7A), and results in a smaller value of maximum ratio (about 2-fold; see Figure 7B) than experimental data (about 2-fold to 2.5-fold). A 3-D extension of the model will avoid such affects, and allows us to study cell clustering inside of a swarm as well as during fruiting body development under starvation [17,18].

We did not quantitatively study the motion of mutants with impaired A motility (A^−^S^+^ mutants) in this paper. As discussed in Results, A^−^S^+^ cells only have persistent active motion when they are within a pilus length of other cells so that the type IV pili can attach to the fibril materials on the surfaces of other cells [34]. Wild-type and A^+^S^−^ mutants both have A motility that can produce persistent active motion. The only difference between wild-type cells and the A^+^S^−^ mutant is the effects of S motility, so it is more convenient to take wild-type cells and the A^+^S^−^ mutant as the modeling systems. By comparing their movements, we could investigate the role of pilus–cell interactions during swarming, which was one of our aims. In Results, we have presented a qualitative analysis of A^−^S^+^ swarming, which demonstrated that the pushing of cells near the swarming edge can explain the expansion of A^−^S^+^ swarms. Preliminary simulations with the pushing mechanism show qualitative agreement with the experiment in terms of peninsula shape and cell ordering (unpublished data). Quantitative modeling of A^−^S^+^ swarming dynamics will require more knowledge of the distribution and mechanical properties of the fibril.

When studying the effects of social interactions, we have related the swarming efficiency with the order of collective motion. This order parameter may provide a novel perspective on quantifying the condition of bacterial swarming. Further experimental investigation of this concept will rely on advances in microscope and image processing of microphotographs. Such experiments require very-high-resolution imaging that can cover large areas of a live bacterial colony [35]. We defined an appropriate order parameter, which characterizes the combined local orientational and positional order. Not limited to the case of myxobacteria swarming, the order parameter provides a quantitative measurement of collective motion in nematic biological systems where local interactions play a dominant role.

We have shown that social interactions mediated by type IV pili, when coupled with active motion, have an alignment effect on neighboring cells and significantly facilitate swarming. Many pathogenic bacteria swarm within infected tissues and have type IV pili as virulence factors. It is likely that the ascent of Proteus mirabilis up the urinary tract is a result of growth and swarming with flagella [36]. Similarly, the spreading of *Neisseria* in infected tissue is related to swarming with type IV pili, since those pili are necessary for virulence [37]. The bacterial swarming model described in the paper may therefore shed light on the colonization and infection process of pathogens.

## Methods

### Modeling the cell body.

In the model, each cell is represented by a flexible string of *N* nodes (Figure 3) consisting of (*N* − 1) segments, each of length *r*. There are (*N* − 2) angles *θ_i_* between neighboring segments. For each cell, we define the following energy function (Hamiltonian):

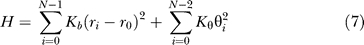



The first term in Equation 7 is the stretching energy determined by the cells' length. The second term is a bending energy. *K_b_* and *K_θ_* are stretching and bending dimensionless coefficients, analogous to the spring constants in Hooke's Law. They determine the extent to which the segment length and angles can change in the presence of fluctuations, respectively. They are the same for all segments and angles. *r*_0_ is the target length of a segment. In our simulations, we choose the number of nodes *N* = 3 (Figure 3), so that *r*_0_ is 2.5 μm (half-cell length). *K_b_* and *K_θ_* are set at 5 and 2, respectively, based on experimental observation that cells do not change their length a lot, but can bend rather easily.

### Modeling cell movement and social interactions.

Let's denote the dark cell in the center of Figure 4 as cell *k*. In the absence of cell–cell collisions, the velocity direction of cell *k* is determined by three contributions: a motility direction, orientation from slime trail, and orientation from type IV pili.

*A motility direction.* The cells secrete slime (polysaccaride) from their tail end, which expands as it leaves the cell body and pushes the cell directly forward [12]. We model this motility by trying to orient the cell along its long axis, which is the tail-to-head direction. The corresponding term in formulae below is denoted as 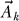 . Small deviations from the direction of long axis are observed [10]. This is modeled using a Monte Carlo reconfiguration algorithm.

*Orientation from slime trail.* When a moving cell encounters a slime trail, it tends to turn through an acute angle to follow the trail. We define a 2-D slime-orientation vector field 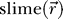 that records the slime trail orientation as a vector assigned to each position 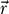 . This vector coincides with orientation of a cell that passed through 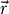 most recently. We make a simplifying assumption of all orientation vectors having unit length. Once a slime trail is laid down at position 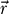 , it will be cleared after the slime aging time *T_s_*_._

*Orientation from type IV pili.* As discussed in an earlier section (Model of cell behavior and social interactions), type IV pilus-mediated interactions are assumed to align neighboring cells. For a particular cell *k*, we average the orientations of its neighboring cells within the pilus-cell interacting area (Figure 4, area I), and define this averaged direction as the contribution of pilus-mediated interactions to the head velocity direction of cell *k*. This term is denoted as 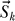
_._

*Cell velocity direction.* When there are no collisions between cell *k* and its neighbors, the direction of its head velocity (denoted as 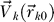 ) is determined by the sum of A motility direction, orientation from slime trail, and orientation from type IV pili:

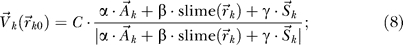
a motility direction:

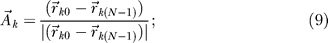
orientation from type IV pili:

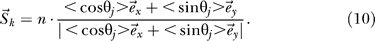



In Equation 8, *C* is a constant cell speed (4 μm/min); *α*, *β*, and *γ* are parameters representing the relative strength of each motility term. Denominators are used in all equations for normalization. The experiments suggest that the forces generated from A and S motilities are nearly the same, approximately 150 pN [9,12]. For an A^+^S^+^ cell, we choose *α* = *γ* = 1.0, and for an A^+^S^−^ mutant, we choose *α* = 1.0 and *γ* = 0. The strength of slime orientation effect is set as *β* = 0.5. Note that the slime-orientation vector field 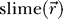 is recorded in a discrete 2-D lattice, with each lattice site having a slime-orientation vector. Slime trails interact with the newly secreted slime, not with the head of a cell. We analyze slime-orientation vectors at the lattice sites covered by the front half of a cell body, and take the direction, which occurs most frequently, as slime-orientation direction to be followed by the cell. It is denoted as slime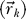 in Equation 8.

Equation 9 determines cell orientation, which is the direction from the tail node (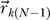 ) to the head node (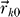 ) and which is considered the A motility direction. In Equation 10, *n* denotes the total number of neighboring cells of the cell *k*. We multiply the expression by the factor of *n* because we think that type IV pili have a stronger effect on the direction of motion of the head node (pili are located at the head of a cell), and that this effect depends on the number of neighboring cells. The terms cos*θ_j_* and sin*θ_j_* are the *x* and *y* components of the orientation vector 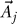 of the *j*-th neighboring cell. These vector components are then averaged (<cos*θ_j_*> and <sin*θ_j_*>) and are taken as the *x* and *y* components of the average direction. 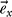 and 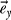 denote unit vectors along the *x* and *y* axes. We model the alignment in such a way that cells orient with their neighbors to the acute angle. That is, if the dot product of the tail-to-head directions of cell *k* and its *j*-th neighbor cell is negative, we choose the opposite direction to 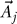 as its orientation. Therefore, we have:

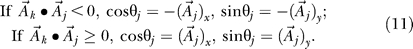



This approach is different from that taken by Vicsek et al. [33]. The alignment is determined through acute angles because we use cell orientations instead of velocity directions.

*Collision-resolving algorithm.* When the head node of cell *k* collides with the body of cell *j*, this collision is resolved as follows:

Calculate distances between the head node of cell *k* and two end nodes of cell *j*;

If one of these distances is less then a cell width, choose at random a new direction such that the dot product of new direction of cell *k* and orientation of cell *j* is positive, and move;

Otherwise, take the average direction of both cells *k* and *j* as the new direction and stall until next time step. (The same method is used in the above alignment algorithm of type IV pilus-mediated interactions.)

*Reversal of gliding direction.* Each myxobacterial cell reverses its gliding direction every 10 min or so. (Reversal periods of myxobacteria follow a distribution with an average of about 10 min.) For simplicity, we choose the reversal periods in accordance with binomial distribution from 5 min to 15 min [38]. Each cell is assigned an inner reversal clock. The initial values of the clock are assigned at random. At each time step of a simulation, the clock value increases by a unit of time. Cell reverses when the clock reaches the value of the reversal period and the clock is reset to zero.

### Simulation of swarming near the colony edge.

*Simulation setup.* The simulation domain is chosen in the form of a rectangle 200 μm by 200 μm (Figure 5). In simulations, a unit length is equal to 0.166 μm and one time step is equal to 0.2 min so that the initial cell length (5 μm) is equal to 30 units of length, and cell width (0.5 μm) is 3 units. As mentioned in the text, we approximate that myxobacteria move at the constant speed of 4 μm/min so that in the simulations, a cell moves a distance of 5 units in each time step.

Initially, cells are distributed within the “Initial Area of Cells” (see Figure 5). Cell centers are distributed at random, but cell orientations are distributed around the radial direction in accordance with the normalized distribution function *f(x)* with a peak at (*π* / 2):

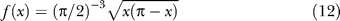



From experimental observations, it follows that a steady rate of swarm expansion is reached only when most cells behind the swarm edge orient themselves outward along the radial direction. Ideally, one would need to choose the initial orientation distribution *f*(*x*) according to the experimental data measured at the beginning of the steady swarming. However, due to the lack of such data, we select the initial orientation distribution function *f*(*x*) in such a form that most cells initially point outward from the swarming edge. Cell growth and division are included in our model as maintaining the average density in the simulation domain near the edge.

*Algorithm implementation.* At each time step, we implement the following sequence of operations for each cell. First, check the inner reversal clock and decide whether to reverse polarity of the cell or not. Then, calculate the velocity direction of the head node according to the model for motility systems. If no collision occurs, move the head node at a distance of five units; otherwise, use the collision-resolving algorithm to resolve the collision. Then, apply Monte Carlo algorithm to reconfigure the positions of other nodes of the cell. Use the procedure suggested in [20]. After moving the head node to a new position, repeat the following operations for (integer part of 2.5*N*) number of steps (*N* is the number of nodes per cell): (i) choose node *i* at random and move it in the direction from node *i* to node (*i* − 1) at a distance of 5 unit lengths; (ii) calculate the energy change Δ*E* due to the relative position change of the nodes. Use the Metropolis algorithm [21] to determine the acceptance probability for the positional change of a node:

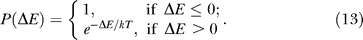
Then, record slime-orientation vectors in the end of individual cell movement at all positions passed through by the cell.

After all cells move, calculate the cell number flux through the boundary into the free space and add the same number of cells into the initial area to keep the cell number in the “Initial Area of Cells” constant. Table 1 provides values of modeling parameters.

### Parameter ranges for the model of slime trail and slime guidance.

Our model depends on two parameters characterizing properties of the slime trail: the slime aging time *T_s_* and the relative strength of slime guidance. In this section, we describe simulation results for different ranges of these parameters to test the robustness of the model.

**Figure 12:**
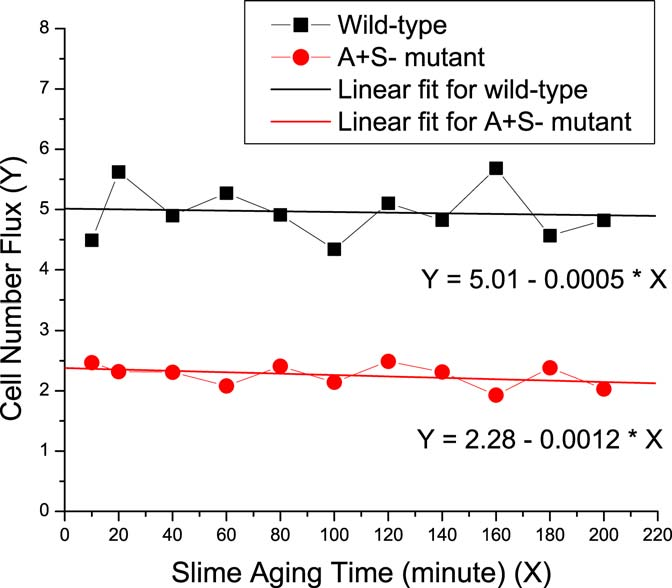
The Dependence of Cell Number Flux on the Slime Aging Time

The slime aging time (*T_s_*) is defined as the lifetime of a slime trail during which it has the ability to guide the motion of a bacteria. We used a value of 20 min in our simulations. In Figure 12, we simulate the swarming of wild-type cells and A^+^S^−^ mutant at the density of 50 K-S units (the same simulation setup as in Figure 6), and varied *T_s_* from 10 min to 200 min (the whole time span of the swarming simulations). We make linear fits for the data points and find that the value of *T_s_* has little effect on simulation results (the cell number flux). This is because slime guidance is primarily a local effect, and slime trails will be washed out by other cells' slimes at short times when the cell density is high. Therefore, the parameter *T_s_* is quite robust for the results in Figure 7B, which is the main validation of our model.

**Figure 13:**
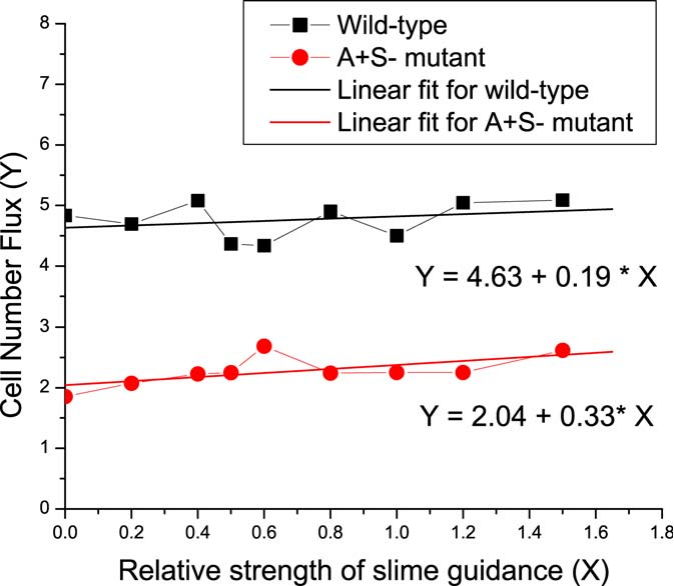
The Dependence of Cell Number Flux on the Relative Slime Strength

The relative strength of slime guidance is modeled by the parameter *β* in Equation 8. We used a value of 0.5 in the simulations (see Methods). Here, we varied *β* from 0 to 1.5, and calculate the cell number flux in swarms of wild-type cell and A^+^S^−^ mutant type at the density of 50 K-S units (the same simulation setup as in Figure 6). The simulation data are plotted in Figure 13 along with the linear fits. We find that as the slime guidance effect gets stronger, the cell number flux increases. It increases slightly faster in the case of A^+^S^−^ mutants than in the case of wild-type cells, with the slopes being 0.33 and 0.19 for A^+^S^−^ mutant and wild-type cells, respectively. This result suggests that as the effect of slime guidance gets stronger, the local alignment of cells and the order of collective motion are both increased. However, this does not affect the results in Figure 7B much, since the increase of cell number flux in the case of A^+^S^−^ mutants is only slightly faster than that in the case of wild-type cells. The ratio of two fitting functions remains greater than 2-fold until *β* = 18.5. This demonstrates robustness of our model with respect to the relative slime strength (see Figure 7B).

## Abbreviations

K-S unit: Klett-Summerson unit
